# Summary of the National Cancer Institute 2023 Virtual Workshop on Medical Image De-identification—Part 2: Pathology Whole Slide Image De-identification, De-facing, the Role of AI in Image De-identification, and the NCI MIDI Datasets and Pipeline

**DOI:** 10.1007/s10278-024-01183-x

**Published:** 2024-07-09

**Authors:** David Clunie, Adam Taylor, Tom Bisson, David Gutman, Ying Xiao, Christopher G. Schwarz, Douglas Greve, Judy Gichoya, George Shih, Adrienne Kline, Ben Kopchick, Keyvan Farahani

**Affiliations:** 1 PixelMed Publishing, Bangor, PA USA; 2 Sage Bionetworks, Seattle, WA USA; 3 Charité - Universitätsmedizin Berlin, Berlin, Germany; 4 Emory University, Atlanta, GA USA; 5 Hospital of the University of Pennsylvania, Philadelphia, PA USA; 6 Mayo Clinic, Rochester, MN USA; 7 MGH/Harvard, Boston, MA USA; 8 Weill Cornell Medical College, New York, NY USA; 9 Northwestern University, Evanston, IL USA; 10 Deloitte Consulting, New York, NY USA; 11 National Heart, Lung, and Blood Institute, National Institutes of Health, Bethesda, MD USA

**Keywords:** De-identification, De-facing, Whole slide imaging, Artificial intelligence

## Abstract

De-identification of medical images intended for research is a core requirement for data sharing initiatives, particularly as the demand for data for artificial intelligence (AI) applications grows. The Center for Biomedical Informatics and Information Technology (CBIIT) of the United States National Cancer Institute (NCI) convened a two half-day virtual workshop with the intent of summarizing the state of the art in de-identification technology and processes and exploring interesting aspects of the subject. This paper summarizes the highlights of the second day of the workshop, the recordings and presentations of which are publicly available for review. The topics covered included pathology whole slide image de-identification, de-facing, the role of AI in image de-identification, and the NCI Medical Image De-Identification Initiative (MIDI) datasets and pipeline.

## Introduction

The Center for Biomedical Informatics and Information Technology (CBIIT) of the United States National Cancer Institute (NCI) convened a two half-day virtual workshop in May 2023 for an unrestricted global audience with the intent of summarizing the state of the art in de-identification technology and processes and exploring interesting aspects of the subject. The workshop was organized in eight sessions over 2 days, each session being focused on a specific topic. This summary provides an overview of the content of the presentations and discussions during each of the sessions. This paper summarizes the highlights of the second day of the workshop, the recordings and presentations of which are publicly available for review at http://wiki.nci.nih.gov/display/MIDI/2023+Medical+Image+De-Identification+Workshop. Being a report of events that transpired, it is not intended to be an introduction to the basic principles of de-identification, but rather to address the state of the art and controversial and promising topics of relevance. Readers interested in background material might want to consult some existing recent comprehensive references [1–3].

A brief summary of the topics presented on the first day includes the following:

- Pathology Whole Slide Image De-identification
  - Introduction to Pathology Whole Slide Image De-identification
  - Anonymization of Whole Slide Images in Histopathology for Research and Education
  - Image DePHI and the Digital Slide Archive: Open-source Tools for Histology Image De-identification
- De-Facing
  - Medical Image De-facing and Clinical Research Data Sharing
  - Face Recognition and De-identification of Research Brain Images with mri_reface
  - MiDeFace: Minimally Invasive De-facing
- The Role of AI in Image De-identification
  - AI’s Ability to Detect Demographics
  - Pixel De-identification Using AI
  - PyLogik: An Open-source Resource for Medical Image De-identification
- NCI MIDI Datasets and Pipeline
  - The Medical Image De-identification Initiative (MIDI)
  - Synthetic Data for De-identification Testing—The MIDI Datasets
  - Building a Cloud-based MIDI Pipeline

## Session 5: Pathology Whole Slide Image De-identification

Whole slide images are images acquired of microscope slides, and even though they are only of a specimen rather than the patient, may contain identifying information. Their format and encoding may present different challenges to radiology images with respect to identity leakage.

### Introduction to Pathology Whole Slide Image De-identification

The session began with an introduction to the topic of whole slide images and their unique de-identification requirements, both for traditional brightfield images, fluorescence images, and other forms of multiplexed tissue microscopy and similar images [4]. Microscopy of tissue sections on glass slides has a long history for diagnosis. These can now be digitized with very high resolution across the whole slide. These offer the opportunity for high throughput automated analysis and AI applications.

De-identification is not often discussed but is no less critical than it is for radiology images. Identifying information may be present in filenames, images of the slide label, burned-in text in the slide area outside the label (including pen marks) whether that be seen only in low-resolution macro images or in the high-resolution scan of the tissue area, and in the image headers, whether the metadata be in public or private tags. A challenge is the broad diversity of file formats and metadata encoding, e.g., Bio-Formats [5] supports 162 different formats, of which over a dozen are from major vendors, with varying degrees of metadata readability. To maintain easy access, these images are encoded as an image “pyramid,” with a baseline very high-resolution image, and then a series of down-sampled images as you go up the pyramid. This allows rapid data access. A number of formats allow for separate pyramids for separate pieces of tissue on the same slide. There may also be a number of associated images, such as label and macro images.

Multiplexed tissue imaging is now performed in research settings seeking to understand the biology of, for example, cancer. These allow for the spatial localization of dozens if not hundreds of proteins from the same tissue section. These result in increasingly larger, higher dimension and higher resolution data. As a case in point, an overview of Sage Bionetworks role as the data coordinating center for the Human Tumor Atlas Network (HTAN) [6] was presented. The HTAN project goal is to construct 3D atlases of the dynamic cellular, morphological, and molecular features of human cancers as they evolve from precancerous lesions to advanced disease. Publicly sharing the whole slide images from this project through the NCI Cancer Research Data Commons (CRDC) [7] nodes, including the Cancer Data Services (CDS) [8] and the Imaging Data Commons [9], necessitated consideration of de-identification of both brightfield hematoxylin and eosin (H&E) and multiplexed tissue images. Even though this is research microscopy data, careful consideration had to be given to data in the context of additional metadata. Specifically, metadata such as “Sectioning days to index,” where sectioning is proximal to imaging, provides an attack vector to reconstruct participant date of birth; this effect is multiplied in the case of longitudinal studies. So date removal during de-identification became important in this project.

Since de-identification in HTAN is performed by the submitting sites, it was necessary to have a centrally managed plan for validation of the site de-identification process. To make this process more efficient, only the header data (TIFF tags), rather than all the pixel data, was streamed from the object store (S3 in this case). Since it was not known a priori what information would be present, all metadata was extracted into key-value pairs. This allowed for access to values of known-to-be-identifying tags (such as dates), as well as unstructured text that was fed into Amazon’s Comprehend NLP service [10], so that values could be ranked by their risk of containing identifiers such as names, which facilitated manual review. Suspicious values were reported back to the submitting centers, who could modify their submission including corrected metadata as necessary.

### Anonymization of Whole Slide Images in Histopathology for Research and Education

The activities of the EcosysteM for Pathology Diagnostics with AI Assistance (EMPAIA) project in this area were presented [11], with reference to a recent publication summarizing this aspect of the project [12]. The motivation was that the fragmented scanner device market introduces a variety of proprietary WSI file formats, which differ significantly in the extents and storage locations of sensitive information. Further, typically, glass slides contain a label with the case identifier, in some cases additionally coded as a 1D or 2D barcode, and during scanning, the label is always captured and stored as an image file in the whole slide image (WSI). Additionally, some scanners perform OCR on these slide labels and/or decode the barcode and store the contained data within the file, among other acquisition-related metadata. When exchanging WSIs, is the partner receiving the data permitted to access this sensitive information? Billing for second opinions requires this data, but for educational purposes or research contributions, there is little need for the sensitive information stored in WSIs. The transmission channel and networking security also have a major impact on data protection, if the sensitive information is present.

Two technical approaches were considered. The first is to separate the tissue image data from the identifying information; this approach needs an interface for accessing the data. The other approach is to remove all identifying information from the WSI (including the associated images with identifiers visible). The case identifier cannot be retrieved, and no access control needs to be implemented. Both approaches are only effective when there is no additional information in the scanned tissue region (e.g., text, pen markings, or even parts of the label).

For this European project, from the regulatory perspective, the GDPR is the legal framework. It contains no specific guidelines, but instead specifies that protection of personal data must ensure a “reasonable” level (which is open to interpretation). It specifies rules for the processing, storing, and sharing of any data identifying a natural person, but does not specifically address health-related and medical data.

From the perspective of GDPR “anonymization,” the digitized tissue itself can serve as an identifier if both the original tissue and its association with the patient are accessible. GDPR considers data to be anonymous if it can only be traced back to specific individuals with a disproportionate effort. From a technical point of view, we can only achieve a so-called “de facto” anonymization (i.e., it is reasonably unlikely that the person subject of the data could be identified) [13]. Extensive manual effort would theoretically allow identification of the case or patient, if the clinical system and either the original slides or alternatively WSIs including the identifier are accessible. However, if such access exists, matching does not add any value, since it is already possible to view the electronic patient record. Thus, if the case ID can no longer be extracted from the WSI, the effort of retrieving patient information may be considered disproportionately large, and thus, the use of the data may be considered unobjectionable from the GDPR perspective.

Examples of metadata and associated images for various vendors’ proprietary file formats were shown. Acquisition-related metadata was illustrated, including dates and times as well as scanner-identifying information, which is also sensitive because in combination with other non-critical data may allow the patient to be identified. Consider a scenario in which WSIs are published as part of an investigation of a rare disease. The label images are removed, but associated metadata is still present within the file. The same scanner serial number of these WSIs can be found in other available WSIs (e.g., from other studies). If those are single-center studies or teaching materials, the WSI may already be assigned to the institute where the patient was admitted. The acquisition date combined with various background information such as study descriptions or appendices can then be used to narrow down the treatment period in more detail. Even though access to the information system would still be crucial, this aggregated information, combined with the rarity of cases of that disease, could drastically reduce the set of eligible patients.

Matters of anonymization policy were also discussed, with five “levels” being proposed. These ranged from (i) simply removing sensitive information from the filename, through (ii) unlinking associated images, (iii) deleting associated images or covering the label prior to scanning (ensuring no ink of the original label is shining through), (iv) deleting sensitive metadata, to (v) dissolving spatial coherence to prevent binary comparison of the pixel data [12]. The de facto anonymization can theoretically be achieved by converting the WSI to DICOM WSI format, and subsequent removal of all sensitive information. However, DICOM is so far only slowly being adopted in digital pathology and not all slide scanner vendors provide anonymization or DICOM conversion. To close this gap, an open-source software library [14] was developed using the C programming language that enables anonymization of WSIs to the level of deleting the label image alongside sensitive metadata within the proprietary file formats. Additional wrappers are provided for Python and JavaScript, as well as a command-line interface tool, and a means of utilizing the library in the browser using WebAssembly. The library is designed to be easily extended to new formats or to updated versions of already existing formats.

### Image DePHI and the Digital Slide Archive: Open-source Tools for Histology Image De-identification

The activities of the cancer Digital Slide Archive (DSA) [15, 16] project related to de-identification were presented [17]. As presented previously, every vendor has their own format. So where in those WSI does the protected health information (PHI) reside? These places are in the filename, in the embedded image metadata, and in the embedded label and macro images. Each scanner normally takes a picture of the label, because without this, you have no idea what the tissue is or the link to the specimen or experiment. A macro image is also captured as a reference to show where the scanned tissue is on the slide. Frequently, the macro image also captures part of the label. Occasionally, the tissue image itself has some questionable text burned in. An example was shown of a macro image that had been publicly released, in which the edge of a label was visible that included the patient’s name and the institution. This example also highlighted that changing the image contrast allowed more text that had been physically redacted to become visible and that handwritten ink marks outside the label were also included. An example of a command-line tool dump of the metadata embedded in the image format was shown, illustrating important metadata such as magnification, which it is desirable to retain, and others that are potential PHI concerns.

A DSA plugin for de-identification was developed, to allow collaborators to process image data that had been loaded into a locally running instance of the DSA. This is available from “http://github.com/dgutman/nci-dsa-deid.” The workflow is designed to minimize user interaction, even though it is not completely automated. The user supplies a comma-separated values (CSV) file or uploads additional metadata to the DSA via an application programming interface (API) to provide a link to the corresponding images before the identifiers are removed, including a mapping to a new filename or case identifier (i.e., pseudonymization). The supplied metadata is validated against a custom JSON schema. The system will not allow redaction if the validation fails. The new metadata can also be encoded in a 2D bar code in a newly generated replacement label image. A graphical user interface (GUI) is provided that summarizes the slides and shows the macro and label images. The slides can be selected by the user for retention of the images or the metadata as-is, or for redaction. Some metadata fields can be automatically retained without bothering the user if they have been predetermined not to contain PHI. Images are staged for redaction as a batch, and then renamed. The user can then review the redacted results and re-run the process if something was missed. The redacted images can then be exported to another target (object store or filesystem). As vendor proprietary formats evolve (e.g., with new scanner models), it is necessary to adapt the default vendor metadata profiles to account for new data elements and add new rules.

More recently, the knowledge gained from experience with using the DSA de-identifier has been incorporated in a new standalone application, ImageDePHI, with support from an NCI Small Business Innovation Research Program (SBIR) grant [18], which is intended to be less customizable but more automated and scalable.

### Discussion

The panelists were asked what the drivers for the development of their software and libraries were. The various projects were intended to diversify the range of different scanners that could be fed into the de-identification pipeline for analysis and automate client-side selection of images without identifying images or information being uploaded. Funding was obtained to address the very large numbers of images being curated for research, to develop the necessary tools, and to develop a knowledge database of metadata that is of concern, or not, to minimize the cost.

It was agreed that it is not necessary to scrutinize every high-resolution tile for burned-in text and that just the overview, top level, and label images are sufficient, assuming that there is not a deliberately malicious attempt to inject content in routine operation.

The matter of participant permission for test images was raised. Also discussed was the difficulty of providing images with identifying information in them to use to test de-identification algorithms.

The negative impact of proliferation of proprietary formats (image format “Whac-A-Mole”) was again emphasized, even those based on nominally standard formats like TIFF, but with proprietary extensions in structured text files including custom XML and JSON rather than private TIFF tags. An example was given of an undocumented JSON text blob present in a TIFF tag containing a value that was a Unix datetime stamp, which would not ordinarily be recognized automatically as identifying information. The option to accept only a restricted range of formats was discussed as a solution. A preference was expressed for there being one accepted format, preferably DICOM, from all vendors and sites in the future. The re-use of the same DICOM PS3.15 de-identification profile [19] for WSI as for other applications was highlighted.

## Session 6: De-facing

It is now well known that medical images of the head and neck that are acquired with sufficient resolution can be reconstructed into visualizations of the face that are near photographic in appearance and pose a risk of re-identification. Modifying the images to remove the information that describes the face is colloquially referred to as “de-facing.”

### Medical Image De-facing and Clinical Research Data Sharing

This session started with an introduction to the speakers in this session and the topics that they would be covering. The first session described the specific de-identification and de-facing requirements for the NCI National Clinical Trials Network (NCTN) and the Imaging and Radiation Oncology Core (IROC) [20]. The volume of data, including images and radiation therapy (RT)-related objects (dose, plans, etc.), required for quality control of RT clinical trials is large, and the intent is to share this data upon trial completion. These require tools and human resources for de-identification. Many of the collections contain brains or head and neck images and related objects, which pose a re-identification risk.

Some of the available de-facing tools that have been previously tested [21] were extended to include the following:

- Quickshear [22], which cuts off facial features beyond a plane
- Biometric_mask [23], which is an aggressive de-facing algorithm that cuts off the face and ears with a rectangular mask
- Carina [24], which initially applies body segmentation and face detection techniques to detect and segment the face, subsequently creating a mask with similar pixel intensities as the face to replace it, and
- mri_reface [25], which matches transformed template and target image intensities and swaps face and ear voxels with transformed template voxels (vide infra)

The tools were evaluated in terms of success rate for preventing re-identification and the maintenance of utility, preserving information required for radiotherapy, specifically changes in radiomic features of critical structures (head and neck organs at risk). The Microsoft Azure Cognitive Face API, a cloud-based service that provides advanced facial recognition and analysis capabilities, was considered but not used due to the restrictive requirements for access. Instead, an open-source package was used [26]. All of the tools tested were successful at reducing the re-identification rate to zero, despite a conservative tolerance factor having been set. Preliminary results suggest that Quickshear and Biometric_mask change the first order radiomics features dramatically, whereas mri_reface has less impact and made Carina change very little. Further evaluations are planned to include more tools, modalities, and cases.

### Face Recognition and De-identification of Research Brain Images with mri_reface

The work of the mri_reface [25] team was presented [27]. The background of public sharing of research data was presented, particularly in the context of Alzheimer’s disease research, the limitations of removing only the identifying text metadata, and the matter of whether a brain MRI, PET, or CT counts as a “comparable image” with respect to the “full-face photographs and any comparable images” clause in the HIPAA Privacy Rule [28]. Motives for re-identification were reviewed.

The face recognition problem was presented as one of the first providing examples of a “training set” of MRI-based reconstructions (rather than photographs) of faces to be recognized, then given a “test” photograph of an unknown individual, and attempting to match it to the correct face to identify them and other accompanying research data. This is a “population-to-sample” attack, which is notably easier than a “sample-to-population” attack (e.g., have an MRI of an unknown individual, and you want to find out from the population of the entire world who might it be). However, the success rates observed in the population-to-sample experiment are sufficient to suggest caution in sharing non-de-faced images. The volunteers, ADNI 3D FLAIR MRI protocol, 3D face reconstruction (using Surf Ice [29] under varying lighting and with various views), and photographic protocol (with five views and gaze positions) for the first 84-participant MRI experiment were described. The Microsoft Azure Cognitive Face API was used. For 83% of participants, the correct MRI was chosen as the #1 match for their photographs [30]. The results were later replicated with PET/CT, showing lesser but still-concerning match rates [31], and Microsoft Azure’s facial recognition performance on the MRI experiment had also improved to as high as 98% for some MR series. Further work suggests that there is a larger risk for MRI 3D than 2D structural sequences and that EPI have minimal risk (8% or less). For comparison, in a prior study using human visual raters for MR, 40% match rates were achieved [32], and with previous-generation automated recognition for CT, 27.5% [33]. Deep learning-based methods have improved face recognition approximately 20 × in the past 5 years [34].

Software to remove identifiable facial features in MRI (“de-facing”) has been available since the mid-2000s but typically has not been applied in US aging studies; because it was believed that re-identification from face recognition would have a low success rate or would not be attempted, there was concern that removing the face would hinder analyses and that existing software had minimal validation (it was assumed that removing at least the lower face, without removing any brain imagery, was good enough). De-facing has generally been more prevalent in European studies and in studies with younger populations. Also, cancer imaging datasets often have additional reasons not to de-face, as the pathology of interest may be outside the brain or face-adjacent.

Algorithms vary in how they modify face voxels, after locating them with an atlas registration. In the past, some algorithms have blurred the outer contour of the face [35], but it has shown that the face can be restored [36]. The presenter’s free, open-source mri_reface tool [25, 37] replaces the face with an average face, rather than removing it, to produce a natural-looking image to reduce effects on pipelines downstream. It uses nonlinear registration, so can more precisely find face voxels than affine approaches. It also replaces ears, teeth, and aliased/phase-wrapped face parts in front or behind the head. It supports T1, T2, T2*, FLAIR MRI as well as FDG, Amyloid, and Tau PET, and CT. In addition, the replacement face matches the noise properties of the input image. The mri_reface performance at preventing recognition was much better than other tools with which it was compared (down to 7%, which still exceeds chance but is better than 97%). The effect of de-facing on regional gray matter volumes was evaluated using several packages, and there was a statistically significant effect, but its magnitude was less than the size of scan-rescan changes. These differences were still considered surprising given that the brain voxels are completely unchanged. Comparisons of the effect of using the algorithm are also available from other research groups [38, 39], which also showed high failure rates, keeping some face or removing some brain for algorithms other than mri_reface and Pydeface. However, Pydeface [40] is not as good at preventing recognition as mri_reface.

Several reasons were presented for why de-facing affects brain measurements even when brain voxels are not modified. Affine registration to a template is affected by the face, especially the nose, and that affects the entire image. The eyebrow ridge is the most important face part for modern face recognition, but it is also within a few millimeters of the frontal lobe, and removing it has the largest effects in the orbitofrontal and frontal poles. Also, Bayesian tissue class segmentations compare relative intensities across entire images, so change in the “not gray matter” intensity distribution affects the probability of being gray matter. Other regions with relatively larger effects include those that are more difficult to segment normally from T1-weighted MRI, specifically the deep gray matter and sensorimotor cortex, which are nowhere near any face that has been replaced.

The latest version of mri_reface includes support for CT and PET and reduces PET recognition rates from 32–41 to 0–3.5%, CT recognition rates from 78 to 5%, and MRI recognition rates from 97–98 to 8%. The effects of de-facing on PET Standardized Uptake Value Ratio (SUVR) are measurable but negligible, being of the same order as effects on T1-weighted MRI gray matter volume and thickness.

De-facing in this manner does not appear to affect correlations with clinical variables in any meaningful way, with high agreement before and after de-facing for correlations with age and cognition. The presenter’s conclusion was that for Alzheimer’s disease imaging research, de-facing with mri_reface had no effect on analyses.

There are limitations of de-facing. Removing or replacing areas outside the brain can limit some secondary re-uses of the data; some applications rely on stereotactic markers on the head, use the shape of the face for placement of leads, or use the complete volume of tissue in the head for radiation dose calibration. For many cancer imaging datasets, de-facing would remove the pathology of interest. Accordingly, future work will measure the effects on re-identification ability of partial de-facing, such as by retaining some coarse face parts or retaining a radius around marked pathology. The eyebrow ridge is the most critical, but it may be possible to retain more of the nose or mouth with only mildly increased risk.

### MiDeFace: Minimally Invasive De-facing

The MiDeFace tool [41] was presented [42]. As already presented, the motivation of developing a new tool is to balance utility and privacy, not to remove too much or too little. For example, removing the entire skull can be problematic if certain corrections on PET need to be performed. The MiDeFace strategy is to first segment the brain and head (including the skull and the eyeballs) to make sure that important things are not removed, then the critical identifying facial features are isolated and removed, including the eyes, nose, mouth, cheeks, chin, ears, and any phase wrap-around in MR. These structures are minimally removed in a way that is hard to reverse. This is performed with a surface-based method.

The segmentation uses Sequence Adaptive Multimodal Segmentation (SAMSEG) [43]. This algorithm segments the whole head, including the skull and the eyeballs, is fast (about 5 min single threaded), and performs intensity normalization and registration to the MNI152 coordinate system. The segmentation identifies important structures to protect from removal.

A surface was built around a binarized average head in MNI152 space; critical features were manually labeled, and a MiDeFace watermark and ripples were added (to make recognition that de-facing has been performed easier). For a set of images from an individual to be de-identified, the head is segmented, binarized, and a surface created around it. Then, so-called “face-bracketing” is performed. The MNI152-edited average face is mapped back into the native image coordinate space, then projected inwards towards the real face, protecting the brain and skull and critical structures, until at least 90 to 95% of the true face is covered. Then, the average face is projected outwards until everything that is outside the true face is also covered. The true face is thus “bracketed” between the inner and outer projections of the average face. The area in between is then replaced with random noise with the same mean and standard deviation as the true data.

In terms of the effect of de-facing on calculations, with 41 subjects using FreeSurfer with and without de-facing to perform cortical and subcortical volume, thickness, and area measurements, there were no situations with significant differences, except that estimated intracranial volume was 0.1% smaller due to a difference in registration.

The program takes less than 8 min to run and has built-in visualization for quality assurance. The tool works on multiple modalities and a mask created on one volume can be applied to another in the same space.

### Discussion

Clarification was sought around which types of PET/CT images were tested with mri_reface, and it was confirmed that the attenuation-corrected PET and CT images from those studies were de-faced separately, i.e., the CT was not used to de-face the corresponding PET images.

Fidelity of segmented regions (in terms of Dice and ICC coefficients for pre-de-faced and de-faced regions and measurements) was high, at least 0.8, often over 0.95. Details are available in the supplementary tables accompanying the referenced papers.

A realistic estimate of the sample-to-population risk for face re-identification was requested for when extracranial pathology requires preservation of the face. This would not be easy to obtain and would require a different data set with special consent and permission to upload to cloud-based facial recognition systems. The availability of databases of photographs, especially of the elderly, is also challenging. Most experiments without corresponding cross-sectional images have been performed by scraping photographs from the Web.

The process of face de-identification is separated from the DICOM header and text de-identification, and then, the de-faced images are rejoined with their de-identified metadata.

The different approaches to quality assurance of de-facing were described for the various tools allowing a human user to quickly visualize that the de-facing has been successful in a matter of seconds.

Potential difficulty identifying the face region for de-facing when only a partial face was present was discussed. One approach is to paste in an average face to complete the dataset and then de-face that.

Whether or not it is necessary to remove teeth and which tools do that was discussed. It was said that this depends on modality (CT versus MR and PET) and distortion (such as susceptibility artifacts).

Beyond de-facing, there is a need for other means of addressing the problem, including more restricted access and data use agreements if the face cannot be removed and the risk is not acceptable.

## Session 7: The Role of AI in Image De-identification

The focus of this session was the use of AI to reduce the risk of sharing data.

### AI’s Ability to Detect Demographics

The session began with an introduction to the ability of AI to detect demographics [44], with reference to work that has recently been published [45], which showed that AI can identify the self-reported race of patients (a social and legal construct), and hence presents a new challenge to de-identification. The technique works across multiple modalities and anatomic regions, is robust to the application of image filters, and has been validated with external data sets. The explanation is not known but may be related to severity of disease and general health in different groups. Modalities and anatomic regions tested included X-ray and CT of the chest, breast, hand, and cervical spine. Experiments were performed to mitigate the recognition, by using saliency maps to guide areas of the image that could be obscured or selectively included; though recognition was reduced, it was still possible. Segmentation of lung from non-lung areas reduced the AUC values but they were still high. Human radiologists perform randomly at this task. Image degradation by blurring, adding noise, or down-sampling reduces performance but signal is still present. The recognition still works even with photographic secondary capture of displayed images. Even when the structure of the image is removed and only the gray level intensity distribution of the pixels is considered, race can still be predicted [46]. Attempts to predict age, sex, and race from other modalities, including echocardiography, have been performed, with better results for sex and race [47]. Another study has shown the ability to predict a discrepancy between thoracic vascular disease occurrence and diagnosis from images, and its relationship to administrative data reflecting linkage to the social deprivation index imputed from geographic location data [48].

### Pixel De-identification Using AI

**Fig. 1:**
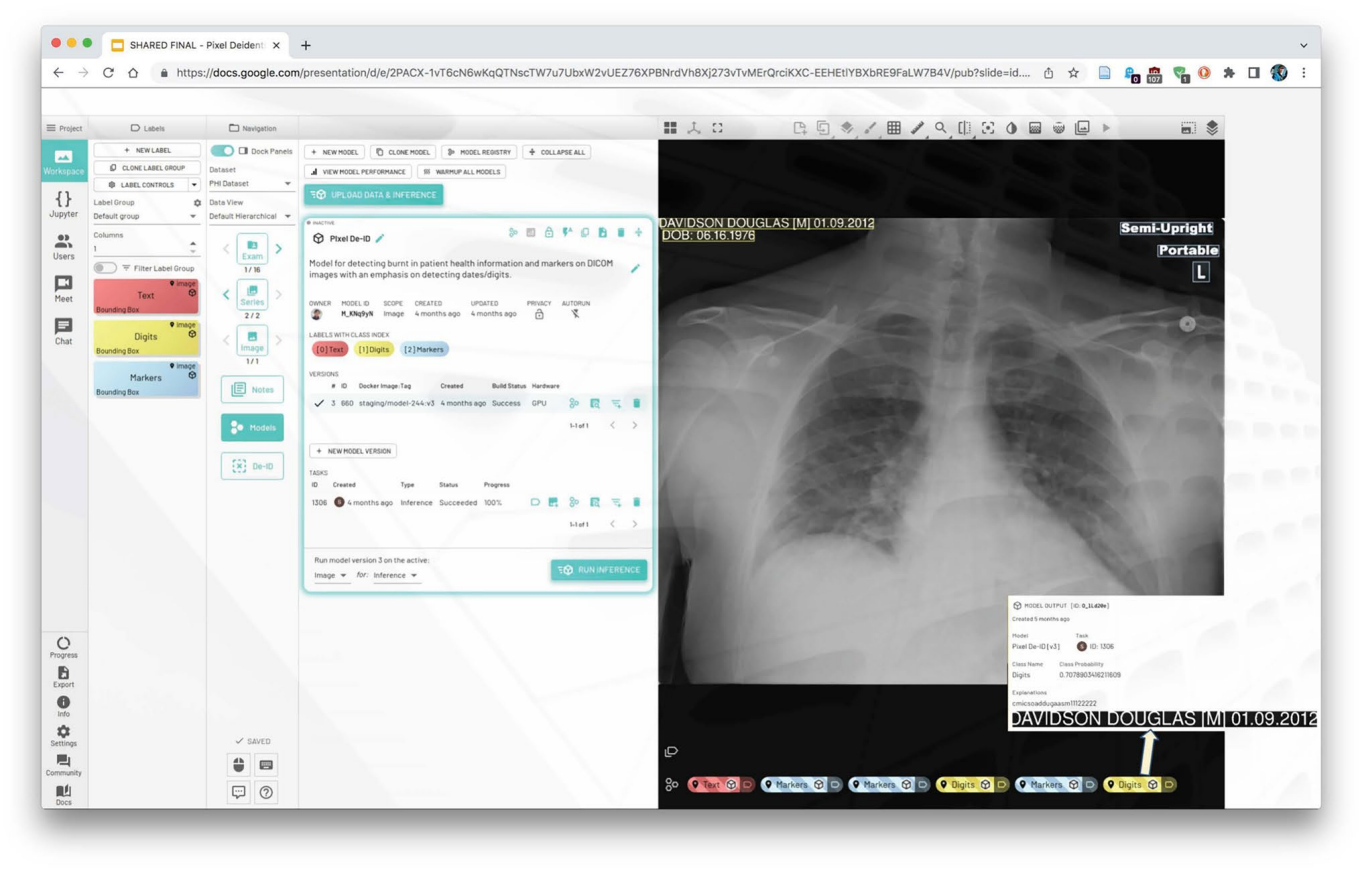
MIDRC Pixel DeID user interface for reviewing the output of the text detection, OCR and classification, and performing redaction

The next presentation focused on the use of AI for burned-in text de-identification in pixel data [49], particularly in the context of image data gathering for the Medical Imaging and Data Resource Center (MIDRC) data repository for COVID data [50]. MIDRC uses many of the tools previously described for The Cancer Image Archive (TCIA) [51] but has recently added an AI-based burned-in text de-identification (Pixel DeID) step, which has also been used by the MD.ai company (Fig. 1).

Examples of a CT dose report screen and an ultrasound image were shown as typical examples of burned-in text identifying information. Rules-based approaches depending on pre-defined locations of identifying information may not always capture the presence and location especially for different manufacturers and models. Burned-in text for other modalities, such as burned-in date and time on chest X-rays, may not be expected and may be very hard to see and easy to miss in a manual workflow. Using automated approaches is not new and has been previously described, but deep learning techniques are improving, potentially making them more useful for this application. Recently, large language models (LLMs) have been introduced, and they may further improve the process. The Pixel DeID workflow includes three steps: detection, optical character recognition (OCR), and classification. The deep learning model classifies the text into text, digits, and radiological markers using a deep learning and named entity recognition (NER) approach. Experiments have also been performed with LLMs such as GPT-4 to improve the output of the OCR step and its classification. The user interface for reviewing the output of the tool and performing redaction (manually or automatically with a script) was illustrated.

A review of the overall workflow was then provided. The RSNA Anonymizer (CTP) [52] is run at the local site; the images are uploaded to a central site where Posda [53, 54] is used for DICOM de-identification, and Kaleidoscope [55] is used for manual review. Then Pixel DeID is run on the data as an additional check before the images are transferred to the MIDRC data repository.

Several examples of the performance of the tool were shown, including the difficult-to-see burned-in date and time in a chest X-ray, which was successfully detected. Some of this work was previously described during RSNA 2022 as part of a MIDRC exhibit.

### PyLogik: An Open-source Resource for Medical Image De-identification

The final presentation in this session [56] described an open-source tool for de-identification, PyLogik [57]. Previous work on burned-in pixel de-identification has had variable rates of success. Existing solutions may have a cost associated with them, are platform-specific, do not batch process, or may result in file size expansion (due to decompression). It may be best to detect all text first and separate the de-identification task. What was described is an AI text detection and recognition algorithm that records text in a CSV file.

The text detector/interpreter consists of convolutional layers (ResNet), recurrent layers (LSTM), and a transcription layer to extract characters in sequence representing words. Several large, openly-available, labeled text image databases were used for training. The tool was demonstrated on ultrasound images with burned-in text.

The process is applied to all frames in an image walking through subfolders in a directory as a batch. The output is saved as JPEG. The simpler task of removing and extracting all text without classifying it is separated from the classification task, which can either be applied to the CSV text information, e.g., by using regular expressions, or all the text can be completely discarded. The compression applied to the images is (visually) lossless JPEG so that the output files are not expanded (are actually smaller), and more can be stored or held in working memory. If a GPU is available, the process uses parallel computing and is significantly faster.

The technique has been validated on several hundred of images and assessed by a clinical expert as 100% effective. The code is modular with de-identification combined with or run separately from cleaning and compression. Various alternative paths were illustrated. In summary, the tool met its initial design constraints and is efficacious, free, operating system agnostic, easy to use, and produces a smaller file size. It works with a variety of data formats, including DICOM, NIfTI, MPEG4, JPEG, and PNG. It suffices for data cleaning for machine learning experiments. It can run behind institutional firewalls. It is applicable to multiple different modalities including ultrasound (+ / − Doppler), CT, MRI, and X-ray. It supports GPU-enabled batch processing and is easy to install and use.

### Discussion

It was asked how often the AI text detection tools found identifiable information that was not detected by humans. Exact numbers were not available but it was reported that this did happen, for example, with burned-in date and time stamps in areas of low image contrast. Clarification was sought on the lossless compression used in PyLogik. The importance of avoiding leakage of identifiable information when using unprotected large language models was emphasized. The issue of recovery of indirect identifiers such as race was discussed, and whether it was individually applicable or only applied to sub-groups. The significance of such hidden signals has not yet been fully elucidated. The usefulness of text extracted from metadata for classifying and selectively filtering extracted burned-in text was discussed. It was noted that PyLogik currently discards the header metadata and retains only the JPEG compressed pixel data and extracted text as CSV. The need for initial on-premise de-identification was discussed, in contrast with the use of cloud-based environments under the control of the source site.

In closing, the question of whether fully automated AI processing for de-identification was feasible yet was asked of the panelists. It was concluded that the state of the art seems to be close to this but that some human quality control process is still likely to be required. Further, there is no absolute test to qualify a process yet. The best that can be done is benchmarking to demonstrate comparable performance.

## Session 8: NCI MIDI Datasets and Pipeline

The focus of this session was the NCI Medical Image De-Identification Initiative (MIDI).

### The Medical Image De-identification Initiative (MIDI)

The session began with an introduction to the initiative [58]. The acronym MIDI initially referred to the initiative but has more recently been used to describe several projects related to the task of de-identification. The establishment of the initiative was motivated by the observation that the demand for sharing of medical images has grown substantially over the past several years coupled with the NIH mandate for data sharing [59]. Scalability and automation of image de-identification must be considered. Furthermore, there has been a general lack of clarity about what level of de-identification is safe and acceptable. The background behind this project included experience with the NCI Imaging Data Commons (IDC) project [9], which does not include a de-identification service but shares already de-identified images in the cloud, and experience with the SBIR image de-identification solicitation [18]. The initiative aims to identify some possible solutions to scalability and automation, as well as specify guidelines on best practices for image de-identification for public repositories.

The initiative consists of a series of interrelated projects to address the following needs:

- A scalable, AI-enabled, image de-identification solution, with reference datasets
- Guidelines and best practices, coupled with community engagement

**Fig. 2:**
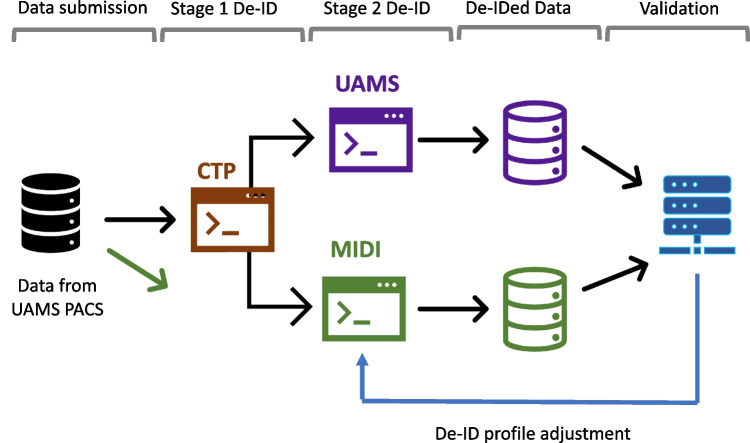
Medical Image De-Identification Initiative (MIDI) Pipeline Phase 3—benchmarking against UAMS TCIA process

Thanks to support for discounted cloud storage and computing from NIH STRIDES [60]. Phase 1 of the MIDI Dataset and Pipeline project included delivery of data with synthetic PHI [61] and use of the Google Data Loss Prevention (DLP) used in the Google Healthcare API for medical image de-identification. In Phase 2, a third-party contractor was introduced to provide unbiased performance evaluation and independent verification of the pipeline performance [62, 63]. In Phase 3, the plan is to test the pipeline with real PHI provided by the University of Arkansas and to benchmark against the TCIA process, which has a solid and reliable track record with NCI image data for more than a decade (see Fig. 2). The goal is to provide a pipeline to which PHI can be uploaded directly to the cloud, given the necessary contractual agreements and security provisions in place. To this end, the necessary NCI IT and cloud security groups have been engaged, with the intent of establishing an Authorization to Operate (ATO).

The final aspect of the initiative is the plan for a MIDI Benchmark Challenge. This will be an opportunity to benchmark the performance of image de-identification tools against a diverse multi-site, multi-modality, reference dataset. It is planned for MICCAI in October 2024.

### Synthetic Data for De-identification Testing—The MIDI Datasets

The next presentation addressed the component of the MIDI program dealing with the creation of datasets that can be used to validate de-identification pipelines [64]. The presentation began by asking the question: how does one validate de-identification tools without violating privacy regulations? Image anonymization algorithms and pipelines must be validated before they are deployed to process data that will be publicly shared. Validation requires robust datasets that can be used in the assessment of de-identification algorithms; this is a common request to the TCIA Help Desk. Synthetic datasets can be constructed to test normal and edge cases and cover all DICOM-defined data object types, and images in non-DICOM formats, informed by experience with actual de-identification processes. Images have been created with both synthetic header data as well as burned-in text in pixel data. The pixel data itself can also be synthesized or known as clean real pixel data. A subset of this data has already been released publicly [61].

The process for creating such datasets involves the following steps:

- Selection of DICOM objects from existing datasets published in TCIA
- Generation of synthetic PHI and insertion into selected DICOM data elements to mimic typical clinical imaging exams
- Using the DICOM Standard and TCIA curation audit logs to guide the insertion of synthetic PHI into standard and non-standard DICOM data elements, in both appropriate and inappropriate places
- Using the TCIA curation tools and procedures to de-identify the synthetic data
- Creating an “Answer Key” to identify what data elements should be modified during a curation process equivalent to that used by TCIA
- Creation of a Python evaluation script to compare the answer key to a de-identified dataset

So far, a total of 172,887 images and other DICOM Instances representing 1448 studies for 1517 synthetic patients have been created. Twenty-eight equipment vendors were represented in the sample. A small sample of synthetic image data was included. PHI was burned into the pixels in some images. Only a small subset has been publicly released, and the remainder sequestered for use in future studies, such as the upcoming MIDI Challenge.

A few examples of unusual places where one finds PHI in DICOM data elements were illustrated, including DICOM standard data elements that should not contain PHI but have been found to do so during TCIA curation. The frequency of occurrence identified in the analysis of TCIA audit logs was indicated. These range from PatientComments, where PHI might be plausible, through RequestedContrastAgent or StationName where it might not be expected. Text strings with PHI have also been found in numeric fields. Private data elements with vendor-defined meanings and values were also considered based on TCIA audit logs and the TCIA knowledge base of private data elements [65].

The “Answer Key” is created from the knowledge of where the synthetic PHI was inserted and a gold standard that is how the TCIA process would handle the inserted PHI using the relevant DICOM PS3.15 profile and options. Where this practice deviates from what is needed in certain situations (e.g., specific projects in specific European jurisdictions), the “Answer Key” can be interpreted accordingly, but serves to indicate which fields require modification.

### Building a Cloud-based MIDI Pipeline

The final presentation in this session described the on-going experience of assembling a cloud-based pipeline for DICOM header and pixel data de-identification using a commercially available service with support from NCI and NHLBI [66]. The solution is cloud-based, since it is anticipated that will be the means of distributing the images. The de-identification methodology focuses on accuracy and is made to conform to the DICOM standard and follow the TCIA processes. This required a customizable pipeline that can both ingest and output DICOM images.

To address the MIDI requirements, Deloitte developed a Google Cloud-based workflow to de-deidentify imaging data and test the performance of the underlying algorithms. This included the following:

- Multi-model image support (CT, MRI, PET, projection X-Ray)
- Processing of DICOM metadata and image-embedded data
- Context awareness, especially in free text fields, to identify research critical data elements and potential PII/ PHI burnt into the image
- Framework to measure performance of workflow with the ability to utilize multiple algorithms developed using in-house tools (e.g., GCP-native vs. externally developed ML/AI-based methods)
- Report with detailed information about de-identified PHI/PII and action taken, and
- A test dataset with synthetic PHI/PII from TCIA used for benchmarking

The MIDI Pipeline technical architecture was reviewed. First, the image is uploaded to a non-de-identified cloud storage bucket. This triggers a pre-processing script that performs quality checks and applies other algorithms necessary. Then, the data is sent to the Google Healthcare API where it is ingested into a DICOM store, which triggers cloud functions to call the pre-configured de-identification API. Once this API completes, the images are transferred to a de-identified DICOM store and then into a de-deidentified cloud storage bucket, as well as exporting results to BigQuery for review and analysis.

The configuration of the de-identification API is flexible. It allows for keeping or removing selected DICOM data elements specified by their tag (name or hexadecimal ID), or grouped by value representation, whether or not to clean text strings and burned-in text, and various text transformation options, including type-specific replacement, and date shifting. Pre-configured profiles matched to those in the DICOM standard can be specified.

The MIDI Pipeline has been tested with multiple datasets to confirm accuracy in de-identification. Two synthetic datasets supplied by TCIA have been run:

- The first contains 1836 DICOM images and an accompanying answer key to validate the pipeline’s work.
- The second dataset contains 23,921 images and was validated by a third party with a TCIA-supplied answer key.

The pipeline is performing at a fast rate of 0.17 s/image average de-identification time for data stored in a single region. It performs with above 98% accuracy per action. It is evaluated for text being retained, text being not null, and text being removed, as well as for dates shifted and pixels hidden. The main remaining issue is the failure to remove text that contains PHI in the more challenging second dataset, where accuracy was only 85%. Examples of successful (true positive) and unsuccessful (false positive and false negative) burned-in text processing were illustrated, including the removal of PHI and the (configurable) retention of text that is not PHI.

Where text failed to be removed or replaced in text strings (false negatives), pre-processing scripts can be used to assist with detection and removal. These include numeric identifiers that are not being removed. Other issues include software versions being mistaken for IP addresses, person names containing underscores or other delimiters not being removed, non-names being mistaken for person names (e.g., when preceded by “MR” misinterpreted as an honorific before a name rather than the abbreviation for magnetic resonance), as well as non-Western and atypical person names. Also, dates occurring in non-date value representation fields may be difficult to detect in the absence of delimiters.

Other miscellaneous issues were encountered. These include creating invalid DICOM values when text strings were replaced with illegal or excessively long values, such as with fixed (placeholder) replacement values or hash values. Also, addresses failed to be replaced when they were not genuine addresses as opposed to synthetic address-like strings (due to Google’s algorithm searching for real addresses). This was an issue for the TCIA-supplied synthetic datasets where the addresses were not real.

In conclusion, the Google Healthcare API DICOM de-identification service shows great promise as a viable option. Further testing is recommended before being deployed in a production environment. Many of the tools used are in Open Beta rather than General Release, and it is hoped that further changes could be made that could improve the pipeline. Automated analysis of pixel removal can be used to identify false positives. Pre- and post-processing can catch many errors currently found. Other solutions can be implemented on top of the Healthcare API and in the cloud to allow other software to be used in the pipeline, such as for de-facing. A human in the loop is still recommended to perform a quality check on all images. Combining the efforts of a human expert and a de-identification service will increase the accuracy (compared to using either alone) and speed up the process.

### Discussion

A question was asked about the potentially different perspectives of regulatory, IRB, and technology transfer folks regarding de-identification. It was emphasized that the focus of the workshop was on technical requirements, not ethical or legal issues. The example of the MIDI Phase 3 project, which involves fully identified data being sent to the cloud to test the de-identification processes and pipelines, was discussed. It has taken more than a year to address the legal and contractual issues involved across multiple groups and institutions, and the agreements and protocols are still not in place. A similar project in Europe took 2.5 years to address the GDPR concerns. The point was made that sending identifiable images to the cloud for such experiments is not that different from deploying a cloud-based PACS for clinical use. Privacy issues are addressed contractually with an expected level of technical safeguards and an appropriate level of trust in the vendor. Vendor audits may be necessary to assure compliance.

A question was asked about preliminary information regarding the Phase 3 project and the use of the off-the-shelf Google solution. It was confirmed that to date, only the Phase 1 and 2 results on synthetic PHI datasets are available. It was emphasized that the experiments to date have involved additional scripts, configuration, and the use of pre-release versions, beyond the off-the-shelf tool. The methodology for testing is generally applicable though. A question was asked about when it would be appropriate to take some other image data from clinical trials, including radiotherapy trials, that might be available through these pipelines, and what is needed in terms of the process to make that happen. It was suggested that we should complete MIDI Phase 3 before expanding it to data from other institutes, centers, projects, and trials. Once the pipeline receives an authorization to operate (ATO), it could be used on other data.

It was confirmed that the current and past MIDI experiments do not address de-facing. This would be a good area to explore in the future. The MIDI Phase 3 dataset does include whole-body PET-CT images, which may include some faces, but it is not the purpose of the experiment to remove them. Further, the MIDI Phase 3 input and output datasets will not be released publicly. The mention of faces digressed into a discussion of the difficulty of detecting images with faces present, as distinct from the removal of faces when they were present.

The matter of the cost of running a de-identification pipeline in the cloud was raised. The MIDI Phase 3 experiment provides an opportunity to estimate the costs of the pipeline, human QC, and the TCIA human-based benchmark. As an example, the cost of the larger dataset previously described for the Phase 2 experiment was approximately $70 USD for a full run on approximately 5 GB of data, not including storage costs or human activities.

The importance of AI in the de-identification process has been made clear, but currently, there is still a need for a human in the loop. The TCIA goal in the long term is to move humans to a purely quality checking role rather than being engaged in every step of the operation. A robust sampling methodology needs to be defined as part of a complete quality management process, as well as focusing on the tasks and subsets that humans are needed for (analogous to triaging).

The next steps towards automation of the process were discussed. The tools need to be improved. Significant changes have already been introduced with deep learning, and more recently, large language models. An appropriate pipeline that is sufficient and accurate needs to be defined and then automated. Lessons can be learned from existing audit logs to provide experience that can be incorporated in improved pipelines and tools. The question was asked as to whether or not active learning could be incorporated in production tools. This needs to be balanced against instability of microservices used. The solution appears to be very close monitoring of performance on an on-going basis to avoid regression. At the same time, there is a desire for continuous improvement. For example, the Google de-identification API does not have fixed versions in the traditional sense, and uses other Google technologies, such as optical character recognition, that continue to be refined and improve accuracy.

There was further discussion about the opportunity to improve the de-identification of clinical trial data, including radiotherapy data, to facilitate public sharing. There is a large amount of data in the radiotherapy pipeline in particular, waiting for a satisfactory de-identification solution.

The question was asked as to what is appropriate to focus AI tools on. A distinction was made between automated handling of routine tasks without an expectation of 100% accuracy on unusual material (such as exotically named celebrity children) and of automated detection of outliers.

## Closing Remarks

The conveners thanked the many contributors to the workshop including the planning group, program committee, session chairs, speakers, panelists, attendees, and support staff. Many of the topics discussed need further development and may serve as future workshop topics in their own right. The role of AI especially, as a tool and a threat, needs to be further considered. They also thanked the collaborators in the MIDI initiative and look forward to reporting on further progress in this respect. The specific topics covered in the second day’s session were briefly summarized. Some action items for continuing the discussion were considered, including the potential establishment of an online forum specific to the subject.
